# Classifications of atherosclerotic plaque components with T1 and T2* mapping in 11.7 T MRI

**DOI:** 10.1016/j.ejro.2021.100323

**Published:** 2021-01-21

**Authors:** My Truong, Finn Lennartsson, Adnan Bibic, Lena Sundius, Ana Persson, Roger Siemund, René In’t Zandt, Isabel Goncalves, Johan Wassélius

**Affiliations:** a Diagnostic Radiology, Department of Clinical Sciences Lund, Lund University, Skåne University Hospital, Medical Imaging Department, Neuroradiology, 22185, Lund, Sweden; b Lund University Bioimaging Centre, Lund University, Klinikgatan 32, BMC D11, SE-221 84, Lund, Sweden; c Cardiology, Skåne University Hospital, Sweden; d Clinical Sciences Malmö, Lund University, Jan Waldenströmsg 35, 91-12, Skåne University Hospital, 20502, Malmö, Sweden; e Department of Radiology, Johns Hopkins University School of Medicine, Baltimore, MD, USA; f Kirby Research Center for Functional Brain Imaging, Kennedy Krieger Institute, F. M. Kirby Center, 707 North Broadway, Baltimore, MD, 21 205, USA

**Keywords:** BSA, bovine serum albumin, HRP, horse radish peroxidase, MRI, magnetic resonance imaging, LRNC, lipid rich necrotic core, IPH, intra-plaque hemorrhage, SD, standard deviation, 11.7T, 11.7 Tesla, TIA, transient ischemic attack, TBS, tris-buffered saline, GE3D, gradient echo three dimensional, CI, confidence interval, CTA, computed tomography angiography, FOV, field of view, ICA, internal carotid artery, ms, millisecond, OCT, optimal cutting temperature, ROI, region of interest, RF, radio frequency, T1w, T1 weighted, T2*w, T2 star weighted, TE, echo time, TR, repetition time, FA, flip angle, 3T, 3 Tesla, 11.7 T MRI, Carotid plaque, Plaque components, Atherosclerosis, T1 maps, T2*maps, Classification

## Abstract

**Background and aims:**

Histopathology is the gold standard for analysis of atherosclerotic plaques but has drawbacks due to the destructive nature of the method. *Ex vivo* MRI is a non-destructive method to image whole plaques. Our aim was to use quantitative high field *ex vivo* MRI to classify plaque components, with histology as gold standard.

**Methods:**

Surgically resected carotid plaques from 12 patients with recent TIA or stroke were imaged at 11.7 T MRI. Quantitative T1/T2* mapping sequences and qualitative T1/T2* gradient echo sequences with voxel size of 30 × 30 × 60 μm^3^ were obtained prior to histological preparation, sectioning and staining for lipids, inflammation, hemorrhage, and fibrous tissue. Regions of interest (ROI) were selected based on the histological staining at multiple levels matched between histology and MRI. The MRI parameters of each ROI were then analyzed with quadratic discriminant analysis (QDA) for classification.

**Results:**

A total of 965 ROIs, at 70 levels matched between histology and MRI, were registered based on histological staining. In the nine plaques where three or more plaque components were possible to co-localize with MRI, the mean degree of misclassification by QDA was 16.5 %. One of the plaques contained mostly fibrous tissue and lipids and had no misclassifications, and two plaques mostly contained fibrous tissue. QDA generally showed good classification for fibrous tissue and lipids, whereas plaques with hemorrhage and inflammation had more misclassifications.

**Conclusion:**

11.7 T *ex vivo* high field MRI shows good visual agreement with histology in carotid plaques. T1/T2* maps analyzed with QDA is a promising non-destructive method to classify plaque components, but with a higher degree of misclassifications in plaques with hemorrhage or inflammation.

## Highlights

• Ex vivo MRI in 11.7 T with T1/T2* maps, is a non-destructive method to study carotid plaque content with good visual agreement with histology.
• Quadratic discriminant analysis on ROI data from T1 and T2* maps, is a promising method to classify plaque content.
• Classification is more challenging in plaques with hemorrhage or inflammation.

## Graphical abstract

**Figure:**
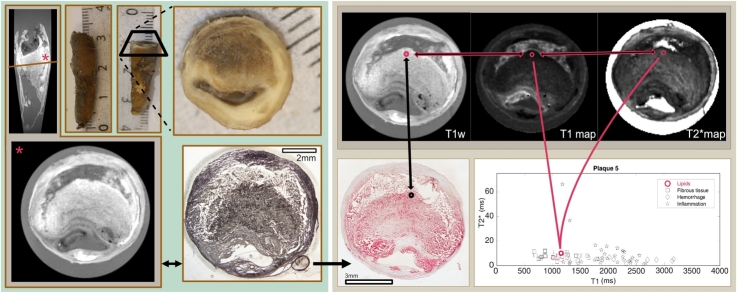

## 1 Introduction

Stroke is the second leading cause of death and the leading cause of adult disability worldwide [1]. A common cause of embolic stroke is atherosclerotic plaques in the carotid artery bifurcation [2]. Morphological characteristics such as intra-plaque hemorrhage (IPH), thin fibrous cap, large necrotic lipid core (LRNC) and cap rupture are associated with elevated risk for embolic stroke [2].

Plaque morphology is commonly studied *ex vivo* with histopathological methods, but there are technical drawbacks with these analyses due to the plaques inherent brittle (calcifications) and loose composition (hemorrhages and necrosis), making freezing, thawing, fixation, sectioning, and cryo-microtome sectioning of the specimen sensitive to distortion of the shape and content of the plaque. There is also inherent loss of tissue during sectioning due to mechanical fragmentation and abrasion.

*Ex vivo* MRI can scan the whole tissue and offers a possibility to study the full plaque volume in 3D. Other advantages of *ex vivo* imaging include the possibility to use long scan times which provides a highly detailed visualization, as well as the possibility to rescan the specimen if needed. High field *ex vivo* MRI scans provide 10-50-micron resolution, which is much higher than *in vivo* scans at 1.5−3 T that typically allows millimeter to submillimeter resolution.

MRI also allows multiplanar reconstructions (MPR) of the acquired scans. In MPR, the observer can change the section plane and adjust it, which greatly improves imaging of complex lesions and facilitates comparison with preoperative imaging or histological specimens.

Furthermore, MRI allows *quantitative imaging* such as T1 and T2* maps based on the T1 and T2* relaxation times for each voxel of the specimen, thereby potentially linking magnetic properties to specific tissue types.

Quantitative imaging based on T1, T2 and T2* maps is well established in *in vivo* cardiac MRI for assessment of the tissue composition such as the amount of scar tissue, edema, and fatty infiltration. Alterations in the T1/T2/T2* values can indicate morphological changes linked to cardiac failure [3,4]. In *in vivo* vessel wall imaging, quantitative imaging using T1 and T2 values have also been linked to disease in the carotid arteries [5,6].

Quadratic discriminant analysis (QDA) is a parametric multivariate classification method often used in supervised machine learning [7], typically to calculate the boundaries between discrete groups and thereby classify the groups [[7], [8], [9], [10]].

High field quantitative MRI with T1/T2* mapping analyzed with QDA can classify plaque content at micron resolution, thereby providing a non-destructive alternative to conventional histological preparation.

## 2 Aim

The aim of this study was to test if high field *ex vivo* MRI (using quadratic discriminant analysis on combinations of T1/T2* relaxation times) could classify carotid artery plaque components detected by histology in specimens from patients with recent TIA or stroke.

## 3 Material and methods

### 3.1 Patients

Between September 2014 and July 2016, acute computer tomographic angiography (CTA) was reviewed for patients with transient ischemic attack (TIA) or Stroke. Patients that met the following inclusion criteria were invited to participate: (i) scheduled for carotid endarterectomy due to ipsilateral TIA or stroke; (ii) age above 18 and ability to give informed consent.

Exclusion criteria was: (i) atrial fibrillation. The study was approved by the local ethic committee and written informed consent was obtained from all patients at inclusion prior to surgery. At surgery, the plaques were snap frozen in liquid nitrogen or fixed with Histochoice (Amresco, Ohio, USA).

### 3.2 11.7 T MRI protocol

The *ex vivo* MRI was performed on an Agilent 11.7 T wide bore vertical magnet (Agilent Technologies, Inc. CA, USA), equipped with a gradient coil with a 1 T/m maximum gradient strength.

Prior to scanning, the samples were placed in an upright position in fresh Histochoice (Amresco, Ohio, USA) for 24 h, to minimize microbubbles on the sample surface that could cause artefacts. The sample chamber was 3D printed (Ultimaker BV, Utrecht, the Netherlands) to fit the scanner and the temperature of the room (20 °C ± 1 °C). Samples were placed in the center of a milli40 probe, a volume coil with an inner diameter of 40 mm, which was used for both transmitting and receiving the MRI signal with quadrature detection. The sample was placed at the center of the volume RF coil to avoid the B1 field variations.

We used a three-dimensional gradient-echo (GE3D) imaging pulse sequence, supplied by the manufacturer as the standard Varian nuclear magnetic resonance VNMR spectrometer software package VnmrJ 3.1 (Varian. Inc. Palo Alto, California, USA).

GE3D generates a 3D image which uses a selective radio frequency (RF) pulse for excitation followed by phase-encoding in two dimensions and readout in the third. The GE3D shim protocol was used to adjust magnetic field homogeneity to minimize image distortions.

The imaging parameters were as follows: field of view (FOV) 30.8 × 15.4 × 15.4 mm^3^, voxel size of 120 × 60 × 60 μm^3^ (interpolated to 60 × 30 × 30 μm^3^ by zero-filling), repetition time (TR) =50 ms, echo time (TE) = 25 ms, flip angle (FA) of 20°, spectral width 100 kHz, 20 averages and scan time of 1 h and 49 min per sample. The protocol above generates T2* weighted images.

The T1 weighted images have the same resolution and positioning as above but with TE = 2.2 ms, TR =18 ms, FA = 20°, with a scan time of 1 h and 38 min.

We used a spoiled 3D gradient echo GE3D sequence to acquire data for the T1 maps with imaging parameters: TR =10 ms, TE = 2 ms, FA = [5, 10, 20, 30 and 40]°, FOV = 30.8 × 15.4 × 15.4 mm^3^ (sagittal orientation for the read direction), actual voxel size = 120 × 120 × 120 μm^3^, interpolated voxel size = 60 × 60 × 60 μm^3^, acquisition time 2 min and 44 s per FA. T1 maps were derived using the variable flip angle method suggested by Blüml et al. [11].

$$S=M_{0}∙\frac{1-\text{exp}\left(-\frac{TR}{T1}\right)}{1-\text{exp}\left(-\frac{TR}{T1}\right)\text{cos}\left(\alpha \right)}\text{s}\text{i}\text{n}(\alpha )∙A)$$

Where $\text{A}=\text{e}\text{x}\text{p}\left(-\frac{TE}{T2*}\right)$ can be normally ignored when TE<<T2*, S is the detected signal, M0 is the equilibrium longitudinal magnetization, TR is the repetition time, and flip angle α.

ImageJ analysis software (National Institutes of Health NIH, Maryland, USA) for data quantifications by using an in-house written plugin was used. We linearized the equation above and constructed a linear equation system to extract the slope coefficient of this linear regression problem. The linearization is performed by multiplying the equation by

$$c_{a}=(1-\text{exp}\left(-\frac{TR}{T1}\right)\text{cos}\left(\alpha \right))/\text{s}\text{i}\text{n}(\alpha )$$

And rearranging and substituting $E=\text{e}\text{x}\text{p}(-\frac{TR}{T1})$, results in

$$\frac{S}{\text{sin}\left(\alpha \right)}=E∙\frac{S}{\text{tan}\left(\alpha \right)}+M_{0}∙(1-E)$$

If we substitute $\frac{S}{\text{t}\text{a}\text{n}(\alpha )}$ with X, and $\frac{S}{\text{s}\text{i}\text{n}(\alpha )}$ with Y. The resulting equation system for each flip angle has a line form $Y_{i}=aX_{i}+b$, where the T1 value is extracted from the slope coefficient of this linear regression problem.

For the data acquisition for the T2* maps, we used the same resolution and positioning as above but TR was 22 ms, TE = [4, 8, 12, 16 and 20] ms, FA = 10°. We used 90° gauss-shaped RF pulses for excitation. Fat suppression was used. Acquisition time was 6 min and 1 s per echo time. T2* maps were derived using the equation:

$$S=S_{0}∙exp(-\frac{TE}{\text{T}2\text{*}})$$

The position of the sample was not changed from one MR acquisition to another.

Images were viewed in ImageJ (National Institutes of Health NIH, Maryland, USA) and the data was quantified in the same program, using an in-house written plugin.

### 3.3 The histological preparation and staining

**Fig. 1:**
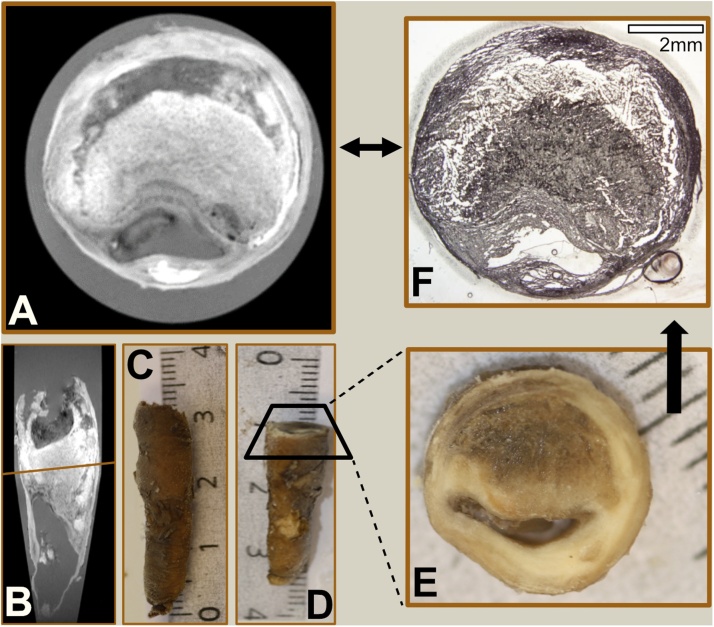
Matching histologic section with axial plane in multiplanar reconstruction. In **(A)** the plaque is seen in the axial plane in MPR on the T1w high resolution image. In **(B)** the plaque is seen from a coronal projection with the line indicating the plane seen on **(A)**. The MRI image volume seen in **(A)** and **(B)** is reviewed in MPR to select the appropriate level for sectioning. In **(C)** the intact plaque is seen prior to sectioning. Guided by the MRI images the plaque is sectioned proximally to the level of interest **(D)**, with the surface seen in **(E)**. The fragment is further sectioned in the cryomicrotome to the level of interest and the final section (prior to staining) is seen in (**F**)*.*

MRI images of each plaque were used to determine levels for sectioning, aiming at regions with large lipid rich necrotic core (LRNC), the highest degree of stenosis and rupture of the fibrous cap (Fig. 1). All plaques contained large calcifications (>1 mm) and in deciding the levels of sectioning, we avoided to cut directly in the calcifications to avoid fragmentation. Regions with smaller calcifications were not excluded when selecting levels of sectioning. The plaque fragments were embedded in optimal cutting temperature (OCT) compound, Scigen, Paramount, CA, USA) and the plaques were cryo-sectioned in transversal 8 μm slices.

For lipid staining, the plaques were fixed with Histochoice (Amresco, Ohio, USA), then dipped them in 60 % isopropanol and then in 0.4 % Oil Red O (ORO) in 60 % isopropanol.

For macrophage staining, the primary monoclonal antibody mouse anti-human CD68, clone KP1 (DakoCytomation, Glostrup, Denmark) was diluted to 1:800 in 1 % bovine serum albumin (BSA) in Tris-buffered saline (TBS) wash, ImmPACT DAB Peroxidase Substrate (Vector Labs, CA, USA) and MACH 3 Mouse HRP-polymer detection (Histolab, Askim, Sweden). Counterstaining was with Mayer’s Hematoxylin.

To visualize erythrocytes in hemorrhage, the primary antibody monoclonal mouse anti-human glycophorin A (CD235a) clone JC159 (DakoCytomation, Glostrup, Denmark) was used, diluted 1:400 in BSA, ImmPACT DAB Peroxidase Substrate (Vector Labs, CA, USA) and MACH 3 Mouse HRP-polymer detection (Histolab, Askim, Sweden). Counterstaining was done with Mayer’s Hematoxylin.

For additional hemorrhage assessment, Perls Prussian blue was also used to stain hemosiderin. Sections were fixated in 4 % Formalin and rinsed in distilled water prior to staining with Perls Prussian blue. Counterstaining was performed with 0.1 % Nuclear Fast Red Solution.

For detection of vascular smooth muscle cells, a primary antibody monoclonal mouse anti-human smooth muscle cells actin clone 1A4 (Dako Cytomation, Glostrup, Denmark) was used, diluted to 1:800 in 1 % BSA stain buffer and TBS wash and ImmPACT DAB Peroxidase Substrate (Vector Labs, CA, USA) and MACH 3 Mouse HRP-polymer detection (Histolab, Askim, Sweden). Counterstaining was Mayer’s Hematoxylin.

### 3.4 Histological image analysis

Prior to staining, every 20^th^ section was scanned with Aperio ImageScope 12.3.2.8013 (Leica Biosystems Inc. Buffalo Grove, IL, USA). Due to lacerations during microtome sectioning, caused by calcifications present in all plaques, it was not possible to match histology with MRI serially. Histologic sections that were damaged or had changed shape, had to be omitted. The histologic sections that were undamaged were matched with the corresponding MRI axial planes visually. The selection of congruent levels between histology and axial planes in MRI was dependent on whether an intact histologic section was attained. A neuroradiologist (MT) compared and matched the scanned sections to the corresponding levels on the T1w MRI in MPR to achieve best visual in-plane congruence (Fig. 1). After staining, the specimens were scanned again, using Aperio ImageScope 12.3.2.8013 (Leica Biosystems Inc. Buffalo Grove, IL, USA). Regions on MRI that visually matched with regions stained for smooth muscle α-actin on histology, were labeled as *fibrous tissue*. Regions on MRI that visually matched with regions stained for ORO, were labeled as *lipids*. Regions stained for CD68 were labeled as *inflammation*. Regions stained by Perls or Glycophorin were registered as *hemorrhage*. Fig. 3 illustrates examples of all 5 different stains that were matched to MRI.

### 3.5 Combined image analysis process

We validated the selection of ROI with histology by using different stains on every level that matched visually between MRI and histology. The stained sections were used to select ROI on the MRI that matched positive stains for the predefined tissue classes.

The process to select ROI was:

(i) The high resolution T1w images were synchronized to the T1 and T2* maps in the axial plane in ImageJ (National Institutes of Health NIH, Maryland, USA), using an in-house written plugin.
(ii) The histological sections of all 5 types of stainings were matched visually with the corresponding level on the T1w high resolution images.
(iii) Following morphologic landmarks on the matched histology/MRI images multiple ROI of 4-9-pixel size were drawn based on positive staining for each of the four labeled tissue groups. Since the T1w image was synchronized to the quantitative T1/T2* maps, the T1/T2*-values- for all ROI were automatically registered.

**Fig. 2:**
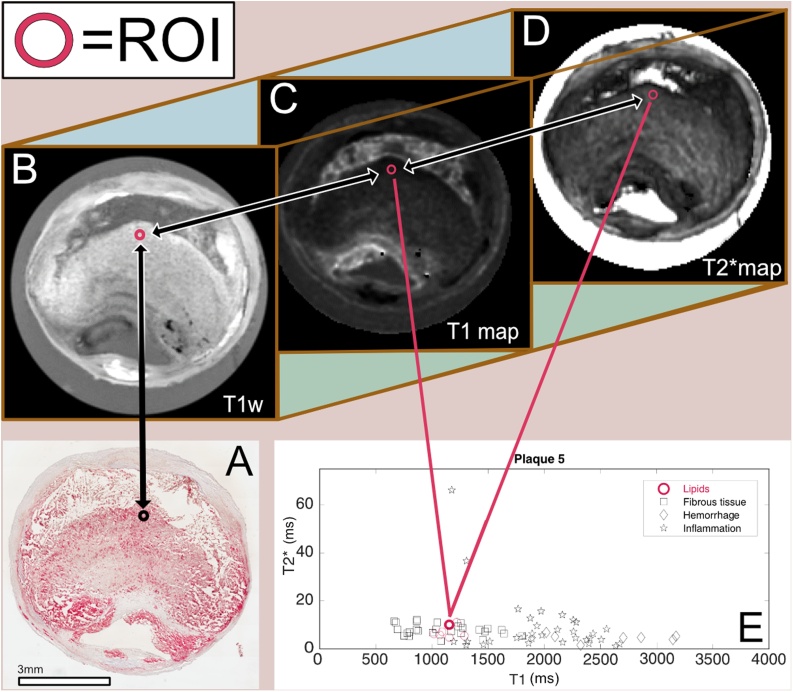
Example of ROI selection validated with histology. Example of the ROI selection process. In (**A)**, a section stained for lipids is matched visually with the corresponding axial plane in the high resolution T1w image (**B**). The high resolution T1w image stack has previously been synchronized with the T1 map and T2* maps (**C)** and (**D)**, so when a ROI (red circle) is selected on the T1w image, the corresponding ROI on the T1 and T2*maps are automatically selected and sampled. The mean T1/T2* values of each ROI are plotted on a graph (**E**) with the T1 relaxation time on the X axis and the T2* relaxation time on the Y axis. In the graph, every red circle represents a ROI selected based on the visually matched histological lipid staining. The process is repeated for all stains and for all plaques (For interpretation of the references to colour in this figure legend, the reader is referred to the web version of this article.).

Fig. 2 illustrates an example of the process of selecting ROI for the lipid group in one plaque and how the T1/T2* values were plotted for each plaque.

**Fig. 3:**
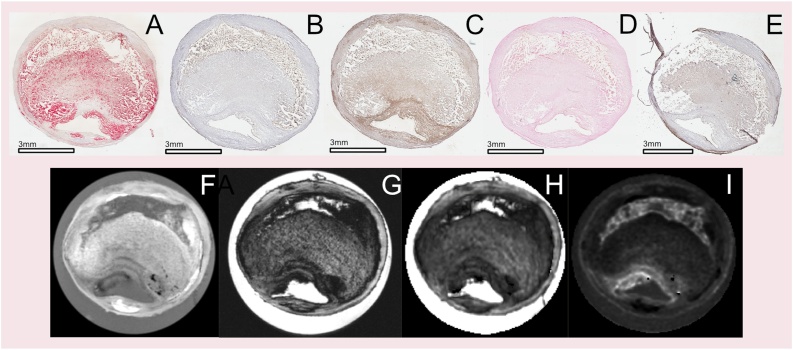
Examples of all 5 different stains with matched level in MRI. The top row shows representative histological images for all five stainings in consecutive sections. Panel (**A)** is Oil Red O staining for lipids. Panel (**B)** is Glycophorin staining for erythrocytes (hemorrhage). Panel (**C)** is CD68 staining for macrophages, corresponding to inflammatory regions. Panel (**D)** is a staining for iron in hemorrhage (Perls, negative in this case). Panel (**E)** is Alpha-Actin staining for smooth muscle cells, corresponding in general terms to fibrous tissue. The bottom row shows the corresponding MRI images. Panel (**F)** shows the high resolution T1w image. Panel (**G)** shows the high resolution T2w image. Panel (**H)** shows the T2* map, and panel (**I)** shows the T1 map (For interpretation of the references to colour in this figure legend, the reader is referred to the web version of this article.).

### 3.6 Statistics

**Fig. 4:**
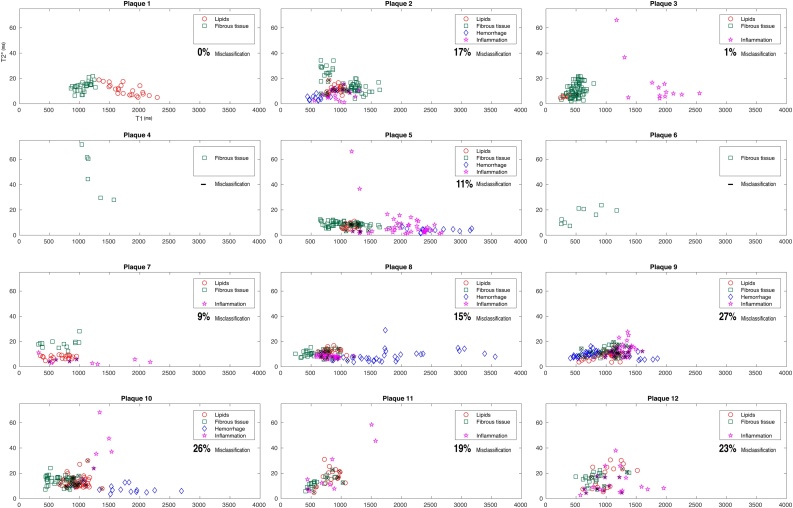
Fig. 4 show the distribution of ROI for the four tissue classes within each of the 12 plaques. The labeled ROI have been visually matched with histology and plotted based on the combination of T1/T2* relaxation times for each ROI. The Graph is shown with T1 value on the X-axis and T2* value on the Y-axis. The ROI that were misclassified by QDA, are marked with an overlapping X and the misclassification rate for each plaque is shown in percentage.

Mean T1- and T2*-times of each ROI where plotted on a graph with the mean T1-time on the X axis and the mean T2*-time on the Y-axis. Every ROI formed one data point on the graph (Fig. 4).

We applied QDA to classify plaque components based on their combinations of T1- and T2*-values and ROI properties.

To classify the four predefined tissue classes with QDA), mean T1 value, mean T2* value, standard deviations for the T1 and T2* value, maximal and minimal T1 values and ROI size (number of pixels) were used as predictor variables for each ROI.

QDA fit an in-dimensional distribution of each labeled ROI to its class: lipids, inflammation, fibrous tissue or hemorrhage, by calculating the mean vector and covariance matrix of each tissue group to determine the center and the shape of ROI distribution respectively. Having fitted the distribution, it is possible to draw boundaries between the tissues by determining were the ROI’s probability is of equal proximity to the center of the group of observations [10]. The QDA was made in Statistics toolbox in MATLAB© (version R2019a, Mathworks, Inc., Natick, MA, USA).

The boundaries are based on the statistical distribution of all the ROI within each tissue type in a three-dimensional fashion based primarily on the mean T1/T2* values and individual ROI’s labeled for a certain tissue group, with parameters that falls under the distribution of another tissue type are judged as *misclassified*. The percentage of misclassified ROI for each plaque is noted in Fig. 4.

## 4 Results

### 4.1 Patients material

During the inclusion period, 46 potential candidates were identified based on clinical data and acute CTA. Of these 46 patients, 15 were accepted for surgery and offered to participate in the study. Two patients chose not to participate. One additional patient accepted for surgery was not operated due to deterioration in clinical status. The remaining 12 plaques from 12 patients were imaged and included in the final analysis. Patient and plaque data are shown in Table 1.

**Table 1:** Patient data.

Number of patients	12
Median age [range]	75 years [63–86]
Sex (M/F)	10/2
Event (TIA/stroke)	6/6
Days from event to surgery (median [range])	12 [5–31]
Days from surgery to MRI (median [range])	52 [9–420]
Fixation at surgery (Histochoice©/Snap frozen)	9/3

### 4.2 Images and classification

During the histologic process, macroscopic calcification fragmented and lacerated the tissue during sectioning in multiple sections but visual agreement of in-plane matching between histology and MRI was accomplished in all plaques, in a total of 70 sections. All 12 plaques contained lipids, areas with inflammation, fibrous tissue, and hemorrhage but only the stained sections matched with MRI were included in the ROI-selection process.

A total of 965 ROIs were analyzed: 407 of fibrous tissue, 250 of lipids, 184 of inflammation and 124 as hemorrhage.

**Fig. 5:**
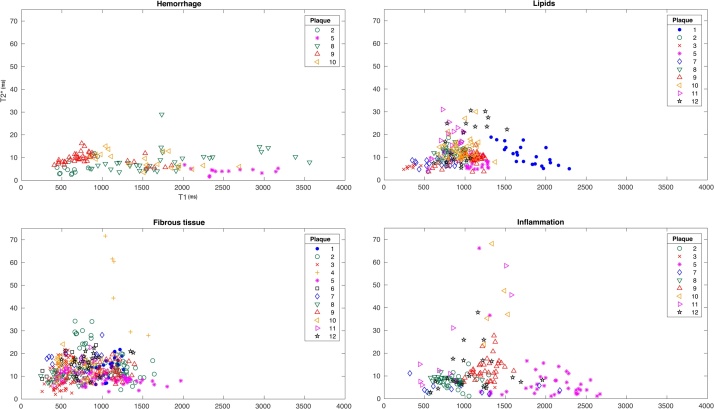
Distribution of ROI for each of the four components in all 12 plaques. ROI for all 12 plaques combined, divided into tissue classes bases. The mean T1/T2* relaxation time are plotted with T1 on the X-axis and T2* on the Y-axis. Every plaque is color and symbol coded according to the side panel seen in each graph. Fibrous tissue was matched between histology and MRI in all plaques. Lipids were matched in 10/12 plaques. Inflammation was matched in 9/12 plaques and hemorrhage was matched in 5/12 plaques. In hemorrhage the range of T1 values is the largest (∼400−3560 ms) and fibrous tissue had the smallest range (∼240−1970 ms) Fibrous tissue has the largest range of T2* values (∼2−70 ms). Lipids and Hemorrhage have equal smallest range of T2* value but Hemorrhage has the smallest if omitting one single ROI with the value 29 ms that seems to be an outlier (∼2−29 ms for Hemorrhage with outlier and ∼2−16 ms without outlier).

Fibrous tissue was matched between histology and MRI in all plaques. Lipids were matched in 10/12 plaques. Tissue with inflammation was matched in 9/12 plaques and hemorrhage was matched in 5/12 plaques (Fig. 5). The largest range of T1 values (∼400−3560 ms) is seen in hemorrhage, and the smallest range (∼240−1970 ms) in fibrous tissue (Table 2).

**Table 2:** ROI data.

*Parameter*	*ROI (n)*	*Min (ms)*	*Max (ms)*	*Mean (ms)*	*Std dev. (ms)*	*Range (ms)*
*Lipids mean T1*	*250*	*241.03*	*2290.56*	*994.98*	*338.93*	*2353.67*
*Inflammation mean T1*	*184*	*317.14*	*2670.81*	*1317.74*	*555.95*	*2049.53*
*Hemorrhage mean T1*	*124*	*406.08*	*3560.94*	*1393.45*	*774.46*	*3154.87*
*Fibrous tissue mean T1*	*407*	*239.36*	*1970.11*	*836.18*	*322.36*	*1730.75*
Lipids mean T2*	*250*	*3.59*	*31.00*	*11.38*	*5.17*	*27.42*
Inflammation mean T2*	*184*	*1.14*	*68.15*	*11.35*	*11.26*	*67.01*
Hemorrhage mean T2*	*124*	*1.55*	*28.98 (16)*	*8.10*	*3.77*	*27.43 (14.67)*
Fibrous tissue mean T2*	*407*	*2.04*	*71.64*	*13.23*	*7.14*	*69.60*

On the contrary, the largest range of T2* values (∼2−70 ms) was seen in fibrous tissue, and the smallest range of T2* values (∼2−29 ms) in hemorrhage. The range may be even smaller since the hemorrhage ROI with the highest value (∼29) might be an outlier. Without the outlier the range of T2* values for hemorrhage is ∼2−16 ms (Table 2).

Fig. 4 shows the distribution of the combination of T1/T2* relaxation times for ROI of lipids, fibrous tissue, inflammation, and hemorrhage, for each plaque.

The plaque with zero misclassification, contained predominantly fibrous tissue and lipids and no ROI for hemorrhage or inflammation that could be matched with histology (Fig. 4). Nine out of 12 plaques had inflammation and/or hemorrhage as well as lipids and fibrous tissue that could be matched between histology and MRI. In these plaques, the mean percentage of misclassified ROI was 16.5 % (CI 11.0–22.0). Two of the plaques contained predominantly fibrous tissue and QDA was therefore not applicable. In Fig. 4, the misclassification rate calculated with QDA for each plaque is shown in percentage and the misclassified ROI are indicated with an overlapping X.

## 5 Discussion

The aim of this study was to evaluate the ability to detect main histological carotid artery plaque components in patients with recent TIA or stroke using quadratic discriminant analysis on the combinations of T1/T2* relaxation times from 11.7 T MRI.

In our study, twelve atherosclerotic plaques from patients with recent TIA or stroke were obtained at endarterectomy on the symptomatic side.

We were able to achieve good visual agreement between histology and high-resolution MRI in all 12 plaques, which is similar to what has been shown in other studies [[12], [13], [14], [15], [16]].

There are several studies where atherosclerotic plaques are studied with MRI to quantify plaque content, at 1,5 T MRI [17,18], 3 T [6,19] or at high field [12,14,16,20]. Itskovich et al. used cluster analysis to quantify plaque components in coronary atherosclerotic plaques scanned in 9.4 T MRI based on multi-contrast high resolution 3D images with good agreement between the cluster-analyzed MRI images and histopathology [16]. Harteveld et al. performed T1, T2, T2* and proton density quantitative imaging of intracranial atherosclerotic plaques *ex vivo* in 7 T MRI, with histologic validation and showed differences in mean T1 relaxation time in-between plaque components such as fibrous tissue, lipids and calcifications, thus demonstrating that different plaque components can be distinguished based on T1 mapping [12]. Coolen et al. applied a 3D T1 and T2 mapping protocol for the carotid artery *in vivo* on six healthy volunteers and one patient with atherosclerosis and showed altered T1 and T2 values in the atherosclerotic plaque. The study was performed *in vivo* in a 3 T MRI system, which rendered lower resolution than in our study (0.7mm^3^ isotropic voxel size, compare to 0.12 mm^3^ isotropic voxel size [6].

In our study, ROI size was small (4–9 voxels), which could be more sensitive to image misregistration. Still, since plaque content was mostly heterogenic and could vary within small regions, we chose not to select larger ROI covering entire plaque component regions since it could introduce a false value by registering the mean value for multiple types of tissue. For example, histology for lipids would not show a large homogenous area with only lipids. Instead, it could be heterogenic, and multiple consecutive histologic stains would show other components localized near lipids. Therefore, we selected small but multiple ROI within an area with a particular composition to overcome the problem with heterogeneity in the tissue.

We found it beneficial to view the T1w and T2*w high resolution 3D MRI in a MPR view when deciding on relevant levels of sectioning. We could thereby alter the view plane to study areas of interest within the plaque, such as rupture sites or lipid rich necrotic cores, prior to histologic sectioning, as well as after sectioning and staining. The MPR facilitated particularly the matching between MRI and histology if the final section on histology was slightly oblique in relation to the center of the lumen.

Our study shows that lipids and fibrous tissue can be classified by combinations of T1 and T2* relaxation times within plaques, although the absolute T1 and T2* relaxation times vary between plaques (Fig. 2). In Coolen et al., the T1 and T2 values showed good repeatability across scan of the healthy volunteers. This could indicate that T1/T2/T2* values are possibly less varied in *in vivo* scans in healthy subjects [6]. The spread of mean T1 and T2* values among plaques in our study could be caused by differences in the handling of plaque tissue at surgical resection and by the differences of time from resection to MRI for each plaque. The difference in time between resection and scan for each plaque may affect plaque content due to the dissolution of loose components. In other studies, the time between surgery and MRI was shorter and more consistent [12,14].

In our study, the wide range of T1 and T2* values of one specific tissue could also in part, be explained by the fact that the maps had voxel size 120 × 120 × 120 μm^3^ (interpolated 60 × 60 × 60 μm^3^), while histologic sections were only 8 μm thick. The matching between MRI and histology done on axial planes only represented a portion of the 120μm^3^ voxel, with a possibility that other tissues, besides the one we wanted to match, could exist within the voxel and contribute to the final T1 and T2* value. Like many symptomatic plaques, the plaques in our study were heterogenic in composition with different types of morphology co-localizing or being in close proximity to each other. This could in part explain the paradoxical result where some ROI would show a longer T1 value for hemorrhage than for fibrous tissue.

In our study design, we did consider matching histologic sections with the corresponding section plane on MRI, automatically and serially, to bypass the matching made by visual inspection. Unfortunately, due to lacerations in multiple histologic sections caused by calcifications, many sections could not be included. Additionally, slight distortion in the histologic section's shape, together with shrinkage (due to histologic processing), could cause a mismatch that made overlaying digital images of histology with MRI very difficult. Due to these facts, the histologic sections were finally visually matched with MRI by an experienced radiologist. Limitations in our study include a small study population and a large time span (9–420 days) between plaque resection and MRI. Another limitation is the fact that we did not focus on calcification, which is a relevant plaque component. The reason is that calcified regions fall off during sectioning if the protocol used aims to stain for other components as lipids and other immunostainings.

In our study we found that classification based on the combination of T1 and T2* relaxation times was more challenging in plaques with hemorrhage and/or inflammation, with higher degree of misclassification.

## 6 Conclusions

11.7 T *ex vivo* high-resolution MRI shows good visual agreement with histology in carotid plaques.

MRI with T1/T2* maps analyzed with quadratic discriminant analysis is a promising non-destructive method to classify plaque content, especially fibrous tissue and lipids. The classification is more challenging in the presence of hemorrhage or inflammation.

In summary, high field quantitative MRI with T1/T2* mapping analyzed with QDA, offers a non-destructive method to image and potentially classify plaque content at micron resolution, thereby providing a potential alternative to conventional histological preparation.

## Ethical approval

All procedures performed in the studies involving human participants were in accordance with the ethical standards of the institutional and national research committee and with the 1964 Helsinki Declaration and its later amendments or comparable ethical standards. Approval (number 472/2005) was granted by the local Swedish Ethical Review Authority.

## Consent to participate and publication

Written informed consent was obtained from all individual participants included in the study.

## Funding

This work was supported by The 10.13039/501100003173Crafoord Foundation#20180610.STINT, The 10.13039/501100001728Swedish Foundation for International Cooperation in Research and Higher Education#IB2018-7532, The 10.13039/501100008590Swedish Stroke Association, Skåne University Hospital, Region Skåne (I-ALF 47447 and YF-ALF 43435) to JW. This work was supported by The 10.13039/501100004359Swedish Research Council, Swedish Heart and Lung Foundation, Skåne University Hospital funds and Swedish Foundation for Strategic Research Dnr IRC15-0067 to IG.

## Authors Contribution

All authors contributed to the study conception, methodology and design. Data collection and data curation was performed by Adnan Bibic, My Truong, Ana Persson and Lena Sundius and René In’t Zandt. Visualization was made by Finn Lennartsson, My Truong and Johan Wasselius. The formal analysis was performed by My Truong and Finn Lennartsson, supported by the late Professor Peter Höglund. Roger Siemund, Isabel Goncalves, Johan Wasselius handled funding and supervision. The first draft of the manuscript was written by My Truong and all authors made critical revisions of the manuscript. All authors read and approved the final manuscript.

## Declaration of Competing Interest

The authors report no declarations of interest.
